# Cost effectiveness of antimicrobial catheters in the intensive care unit: addressing uncertainty in the decision

**DOI:** 10.1186/cc7744

**Published:** 2009-03-11

**Authors:** Kate A Halton, David A Cook, Michael Whitby, David L Paterson, Nicholas Graves

**Affiliations:** 1 The Centre for Healthcare Related Infection Surveillance & Prevention, GPO Box 48, Brisbane, Queensland, 4001 Australia; 2 Institute of Health & Biomedical Innovation, Queensland University of Technology, 60 Musk Avenue, Kelvin Grove, Queensland, 4059 Australia; 3 Intensive Care Unit, Princess Alexandra Hospital, 199 Ipswich Road, Woolloongabba, Queensland, 4102 Australia; 4 Infection Management Services, Princess Alexandra Hospital, 199 Ipswich Road, Woolloongabba, Queensland, 4102 Australia; 5 University of Queensland, Royal Brisbane & Women's Hospital, Butterfield Street, Herston, Queensland, 4029 Australia

## Abstract

**Introduction:**

Some types of antimicrobial-coated central venous catheters (A-CVC) have been shown to be cost effective in preventing catheter-related bloodstream infection (CR-BSI). However, not all types have been evaluated, and there are concerns over the quality and usefulness of these earlier studies. There is uncertainty amongst clinicians over which, if any, A-CVCs to use. We re-evaluated the cost effectiveness of all commercially available A-CVCs for prevention of CR-BSI in adult intensive care unit (ICU) patients.

**Methods:**

We used a Markov decision model to compare the cost effectiveness of A-CVCs relative to uncoated catheters. Four catheter types were evaluated: minocycline and rifampicin (MR)-coated catheters, silver, platinum and carbon (SPC)-impregnated catheters, and two chlorhexidine and silver sulfadiazine-coated catheters; one coated on the external surface (CH/SSD (ext)) and the other coated on both surfaces (CH/SSD (int/ext)). The incremental cost per quality-adjusted life year gained and the expected net monetary benefits were estimated for each. Uncertainty arising from data estimates, data quality and heterogeneity was explored in sensitivity analyses.

**Results:**

The baseline analysis, with no consideration of uncertainty, indicated all four types of A-CVC were cost-saving relative to uncoated catheters. MR-coated catheters prevented 15 infections per 1,000 catheters and generated the greatest health benefits, 1.6 quality-adjusted life years, and cost savings (AUD $130,289). After considering uncertainty in the current evidence, the MR-coated catheters returned the highest incremental monetary net benefits of AUD $948 per catheter; however there was a 62% probability of error in this conclusion. Although the MR-coated catheters had the highest monetary net benefits across multiple scenarios, the decision was always associated with high uncertainty.

**Conclusions:**

Current evidence suggests that the cost effectiveness of using A-CVCs within the ICU is highly uncertain. Policies to prevent CR-BSI amongst ICU patients should consider the cost effectiveness of competing interventions in the light of this uncertainty. Decision makers would do well to consider the current gaps in knowledge and the complexity of producing good quality evidence in this area.

## Introduction

Catheter-related bloodstream infections (CR-BSIs) increase health costs and patient morbidity [1], and their prevention has been the target of national initiatives to create safer and more efficient healthcare systems [2,3]. These healthcare-acquired infections are among the group for which the US Centers for Medicare and Medicaid Services are now able to withhold payments [4], thereby shifting the cost onto the hospitals rather than healthcare payers who reimburse the clinical facilities. Given this change in the economic context for infection control, decision makers are likely to pay more attention to the cost effectiveness of interventions they employ to reduce rates of CR-BSI [5].

The use of specific types of antimicrobial-coated central venous catheter (A-CVC) to prevent CR-BSI has been shown in earlier economic evaluations to be cost-saving and generate health benefits within the wider healthcare system [6,7]. However, not all have been evaluated and there are concerns over the quality of these evaluations and the usefulness of their findings for real-world decision making [8].

Problems with the existing economic evidence contribute to the ongoing uncertainty about the use of A-CVCs. First, the relative cost effectiveness of the different types of A-CVC is unknown as none of the previous evaluations compared all available types. Second, recent epidemiological evidence [1] suggests earlier evaluations may have overestimated the attributable mortality and length of stay associated with CR-BSI, and these were key drivers of the results [8]. Third, the excess length of stay due to infection is a major source of cost savings and the dollar value given to each bed day released will depend on the preferences of the decision maker. They cannot be directly observed and require careful elicitation, and the valuation may change depending on who is making the decision. To date there has been no discussion as to how these value judgments are derived, creating another subtle source of uncertainty in the results of the earlier evaluations.

There is continued uncertainty among clinicians over which, if any, A-CVC to use. Clinical guidelines recommend their use only in specific circumstances [9], and evidence suggests that the uptake of these technologies remains patchy [10,11]. The purpose of this study is to evaluate the cost effectiveness of adopting A-CVCs to prevent CR-BSI in Australian intensive care units (ICUs). We considered all available catheter types, used updated estimates of the consequences of infection, and explored how uncertainty can impact the adoption decision. By doing so, we provide a deeper analysis of this infection control decision that will support those working in this clinical area.

## Materials and methods

We undertook an economic evaluation to identify the cost effectiveness of triple-lumen A-CVCs for standard use in Australian adult ICUs. We considered all commercially manufactured A-CVCs sold in Australia: minocycline and rifampicin (MR)-coated catheters; silver, platinum and carbon (SPC)-impregnated catheters; and two chlorhexidine and silver sulfadiazine-coated catheters; one coated on the external surface (CH/SSD (ext)) and the other coated on both catheter surfaces (CH/SSD (int/ext)). The baseline comparator was uncoated polyurethane catheters.

### Model development

**Figure 1:**
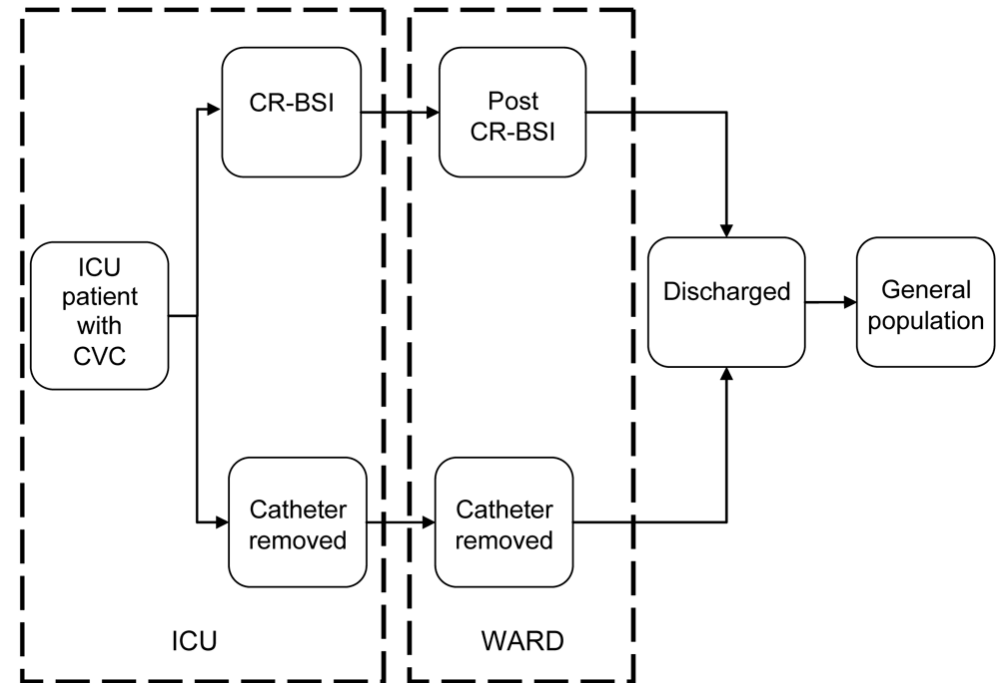
Markov model used for the evaluation.

Clinical events used to structure the model were identified in conjunction with intensive care clinicians. Clinical and economic events under a healthcare perspective were identified and organized into Markov states (Figure 1). Patients were assumed to receive a CVC on entry to ICU, and over subsequent daily cycles either retained their catheter, had it removed, or developed a CR-BSI [12]. Patients faced an underlying risk of mortality whilst in the ICU and a further risk should they develop CR-BSI. The surviving cohort was modeled for the remainder of their lifetime in monthly cycles, moving to yearly cycles 1 year after discharge.

ICUs were assumed to have existing optimal infection control procedures in place. Multiple catheterizations, catheters inserted or removed outside the ICU and future catheterizations were excluded. We did not model catheter colonization, as this event alone carries no health or economic consequences, or anaphylactic reaction to the CH/SSD catheters [13], as this event is rare. The effectiveness of all catheters and the consequences of CR-BSI were considered independent of patient age or disease severity and causative microorganisms. Treatment success was considered to be final and we did not model recurrence of infection. Economic costs were measured in 2006 Australian dollars and health outcomes in quality-adjusted life years (QALYs). Costs and health outcomes relating to the original ICU experience but occurring in future time periods were discounted at a rate of 3%. In line with recommendations [14] we did not attempt to model future access to healthcare.

### Framework for evaluation

The strong evidence that CR-BSI increases of length of stay in the ICU and general wards suggests that health care costs will vary between catheters if they differ in effectiveness at preventing infection. Conversely, there is relatively weak evidence for the causal relationship between CR-BSI and mortality; this implies a tenuous difference in health outcomes for different catheter choices. One approach is to assume that health outcomes (measured in QALYs) are the same for all catheter types and so economic evaluation could be simplified to a cost-minimization analysis. This approach to making decisions is, however, unhelpful [15]. Studies have not shown an absence of effect; rather, they have been unable to show a statistically significant positive effect between CR-BSI and mortality. The best interpretation is that we are uncertain about any relationship between CR-BSI and mortality. Thus we chose to use cost effectiveness analysis (CEA) and explore the impact of the uncertainty about attributable mortality (and other model parameters) on our conclusions.

### Data sources

Parameters used in the model are shown in Table 1. Where estimates were obtained from the literature, relevant articles were identified via reproducible searches in the Medline database to 1 January 2008, and earlier economic evaluations of strategies to prevent CR-BSI were reviewed. Bibliographic details for all relevant studies identified in these searches are provided in Additional data file 1.

**Table 1:** Parameter estimates used in the model

**Parameters**		**Baseline estimate**	**Variation (SEM)**	**Distribution**	**Source**	**Level of evidence**
Infection-related events:						
Probability of CR-BSI		Modeled in stepwise increments^a^		Beta	Database	1
RR mortality (CR-BSI)		1.06	0.18	Log normal	[1]	2
Extra days in the ICU		2.41	0.83	Log normal	[22]	2
Extra days on hospital ward		7.54	1.81	Log normal		

Effectiveness A-CVCs (RR):						
SPC		0.54	0.62	Log transformed normal	[21]	1 +
CH/SSD (ext)		0.66	0.17			
CH/SSD (int/ext)		0.70	0.43			
MR		0.39	0.43			

Baseline probabilities of mortality:						
ICU mortality		0.098	0.002	Beta	Dataset	2
Hospital mortality		0.069	0.001	Beta	Dataset	2
Annual mortality post discharge	Year 1	0.050	0.002	Beta	[24]	2
	Years 2 to 3	0.027	0.002			
	Years 4 to 5	0.028	0.002			
	Years 6 to 10	0.037	0.003			
	Years 11 to 15	0.042	0.003			
Underlying annual mortality	45 to 64 years	0.004	-	NA	[25]	1
	65 to 84 years	0.030	-			
	85 + years	0.140	-			

Utilities:						
Utility ICU		0.66	0.27	Beta	[28]	3
Utilities population norms	50 to 59 years	0.80	0.22	Beta	[27]	3
	60 to 69 years	0.79	0.19			
	70 to 79 years	0.75	0.25			
	80 + years	0.66	0.29			

Costs, 2006 AUD:						
ICU bed day		3,021	-	NA	[31]	4
Hospital bed day		843	-	NA	[32]	3
Diagnostics CR-BSI		101.70	-	NA	Database	1
Treatment CR-BSI		591.30	-	NA	Database	1
Additional cost per catheter	SPC	22.36	-	NA	Database	1
	CH/SSD (ext)	11.64	-			
	CH/SSD (int/ext)	41.35	-			
	MR	59.36	-			

^a^Available on request from the authors.

A-CVCs, antimicrobial central venous catheters; CH/SSD, chlorhexidine silver sulfadiazine; CR-BSI, catheter related bloodstream infection; ICU, intensive care unit; int/ext, internally and externally coated; MR, minocycline and rifampicin; RR, relative risk; SPC, silver, platinum and carbon.

The context of the evaluation was a level 3 (tertiary referral) ICU [16]. Based on a 4-year dataset of 11,790 ICU admissions we assumed that 17% would receive a CVC [17]. This catheterized cohort had a mean age of 62.7 (standard deviation (SD) 17.2) years, mean Acute Physiology and Chronic Health Evaluation II score of 17.1 (SD 8) and 65% were male. These estimates are comparable to those reported for 46 publicly funded ICUs by the Australia and New Zealand Intensive Care Society [18]. Baseline risk of ICU mortality was 9.8% and 16.1% by hospital discharge.

Probability of CR-BSI was modeled as increasing in stepwise increments with duration of catheterization [19] to give an overall incidence of infection of 2.5%. This was observed in routine surveillance data collected from February 2001 to December 2005 in 21 medium-to-large public hospitals in Queensland, Australia [20]. Estimates for the effectiveness of each type of A-CVC were taken from a single systematic review, chosen from amongst 14 identified because it provided relative risks separately for each type of coating [21].

The relative risk of hospital mortality associated with CR-BSI was estimated to be 1.06 [1]. Given a 9.8% baseline risk, this corresponds to an absolute increase in mortality of just under 1%. Excess length of stay due to infection was estimated at 2.4 ICU and 7.5 general ward days [22]. These values were chosen from amongst 19 estimates of attributable mortality and 11 estimates of increases to length of stay identified in a literature search, as they were of high quality (judgment based on Samore and Harbarth [23]) and the population was comparable to our ICU context.

Annual mortality rates for 15 years post ICU discharge were taken from a data linkage study [24] that followed over 10,000 Australian ICU patients. Subsequent life expectancy was based on Australian Institute of Health and Welfare published age-specific mortality rates [25]. To calculate QALYs, preference based utility weights were assigned to cycles spent in the ICU and 6 months immediately post discharge. Although evidence suggests that quality of life may be reduced in some survivors for a longer period post discharge [26], information on this was unavailable for our population. Therefore, to be conservative, life expectancy for those surviving beyond this period was adjusted using Australian population quality of life norms [27]. In all, 14 studies estimated utility weights for ICU patients. Values were used from the study [28] with participant demographics most similar to our cohort. This study used an instrument (the EQ-5D) shown to predict weights similar to the Australian Quality of Life instrument used to derive population norms [29]. No further quality of life decrement was attributed to CR-BSI.

All costs were valued at 2006 prices, using the Australian Bureau of Labor Statistics Consumer Price Index [30] to adjust where necessary. Consumable costs in the evaluation included the price of a catheter, diagnosis costs of one catheter tip and two blood cultures per CR-BSI and treatment costs. Treatment costs were a weighted average of the cost of standard regimens for causative organisms observed within the surveillance system: 2 weeks vancomycin, 10 days ticarcillin, 4 weeks fluconazole. Prices for all consumables reflect those faced by Queensland Health decision makers.

The economic value of bed days released by the prevention of CR-BSI was assessed from two alternate perspectives. A broader perspective of the healthcare decision maker who manages waiting lists, and for whom there is a real economic benefit in releasing a bed day for another patient to occupy, and a narrower perspective of a manager working within an ICU or hospital. Values to represent the broader perspective were obtained for an ICU bed day from a detailed costing study of an Australian ICU [31] and for a general ward bed day from an earlier economic evaluation which considered spending patterns for Australian public hospital services [32]. These estimates of AUD $3,021 and AUD $843 represent short-run average costs calculated by dividing total costs (that is, fixed and variable costs) by the total bed days for a 12-month budget period. They may or may not approximate the economic opportunity cost of losing a bed day to CR-BSI. The alternate narrow perspective value considered only the variable cost per bed day. Variable costs are the cash savings that budget holders within the hospital can recoup if bed days are not used; they include items such as fluids, dressings and pharmaceuticals. These costs are meaningful to hospital administrators, who cannot avoid fixed operating costs even if infections reduce [33]. An important caveat for the narrow perspective costs is that they decrease over the duration of ICU stay [34]; we assumed it would be later, less costly, days released by preventing infection and adjusted our baseline estimates based on the daily pattern of variable costs reported in a similar patient population [34], to give estimates of AUD $335 for ICUs and AUD $101 for general wards.

### Model evaluation

Model evaluation was performed in three stages. First, uncertainty in the cost effectiveness evaluation was ignored and a single value was used for each model parameter. The incremental change in costs (Δ*C*) and QALYs (Δ*E*) were estimated for each type of A-CVC and incremental cost effectiveness ratios (ICERs) were calculated (Δ*C*/Δ*E*). A catheter type was considered to be cost-saving if greater health benefits and reduced costs were achieved as compared to uncoated catheters, and considered cost effective if the ICER was below a threshold willingness-to-pay ratio (λ) of AUD $40,000 per QALY. This threshold was chosen based on an analysis of positive reimbursement decisions made by the Pharmaceutical Benefits Advisory Committee, Australia [35].

Second, probabilistic sensitivity analysis (PSA) [36] was used to capture uncertainty in the current data. The error in each estimate (parameter) used in the model, as described by its standard error, was characterized using an appropriate probability distribution (Table 1), except costs that were assumed known in the context of the evaluation. A total of 10,000 Monte Carlo simulations were run; in each one a new value was drawn for each parameter from within the distribution specified. The results of each simulation were presented as the monetary net benefits generated by each catheter type. Monetary net benefits were used as they are linear and have improved properties as compared to ICERs for decision making [37]. Although we expressed net benefits in monetary terms, this is not a cost-benefit analysis. Monetary net benefits were calculated by valuing incremental QALYs generated by the A-CVC at $40,000 each (the willingness-to-pay threshold) and then subtracting incremental costs (that is, NB = (λ × Δ*E*)-Δ*C*).

The average monetary net benefit across the 10,000 simulations was calculated for each catheter type along with 95% confidence intervals (CIs). Given the economic objective of maximizing benefits given scarce resources, the optimal decision was defined as the catheter associated with the highest average monetary net benefit; choosing anything else would incur an opportunity cost. The likelihood of error in this conclusion was also calculated. The proportion of simulations in which a catheter returned the highest monetary net benefit represents the probability that catheter type is optimal; 1 minus this proportion represents the probability that the catheter does not return the highest monetary net benefits, but instead incurs a cost, and the decision is incorrect. Table 2 illustrates this interpretation using hypothetical data for two novel treatments compared to standard practice.

**Table 2:** Monetary net benefits for a hypothetical evaluation comparing two novel treatments to standard practice

	**Standard practice**	**Treatment A**	**Treatment B**	**Optimal choice**
Simulation 1	140	150	160	B
Simulation 2	100	110	120	B
Simulation 3	110	100	100	Standard
Simulation 4	100	150	130	A
Simulation 5	130	120	110	Standard
Average expected net benefit	116	126	124	Standard/A/B = 40%/20%/40%

Results are expressed as monetary net benefits, each simulation is equally likely to be 'true'. Treatment A is associated with the highest expected net benefit (AUD $126), but because the distribution of monetary net benefits is skewed, it is preferred in only 20% of samples. Treatment A is therefore optimal, but the error probability associated with this choice is 80%. This probability is substantially higher than the 5% used for tests of statistical significance. The choice to remain with standard practice still carries a 40% probability of not returning the highest monetary net benefits and could be expected to incur economic costs of AUD $10 (AUD $126 minus AUD $116). The alternative with the highest monetary net benefit is the optimal decision, but that decision can be highly uncertain.

Third, we used scenario analysis to explore uncertainty introduced by the fact that some data used in the model was of low quality. Using a modified version [38] of the potential hierarchies of data sources for economic evaluations [39], we identified data of medium and low quality (Table 1); defined as scoring level 3 or below. For each parameter with medium/low quality data we assigned a plausible alternate value and re-evaluated the model. The dollar value given to the opportunity cost of bed days was changed to reflect the broader and narrower perspectives of different decision makers. A higher estimate for attributable mortality of 15% was used, which is comparable to that assumed in earlier economic evaluations of A-CVCs. Higher estimates of the extension to stay of 6.5 days on the ICU and 6 days on the general ward due to CR-BSI were used. All utility weights for health states were removed, which is equivalent to using unadjusted life years rather than QALYs. The final scenario reflected the fact that the absolute effectiveness and cost effectiveness of A-CVCs will be dependent on starting rates of infection [40]; lower (0.8%) and higher (5.0%) rates were used to cover the range reported from individual hospitals within the surveillance dataset. In each scenario we reran an analysis to recalculate the monetary net benefit for each catheter type and so identify the optimal catheter.

## Results

**Figure 2:**
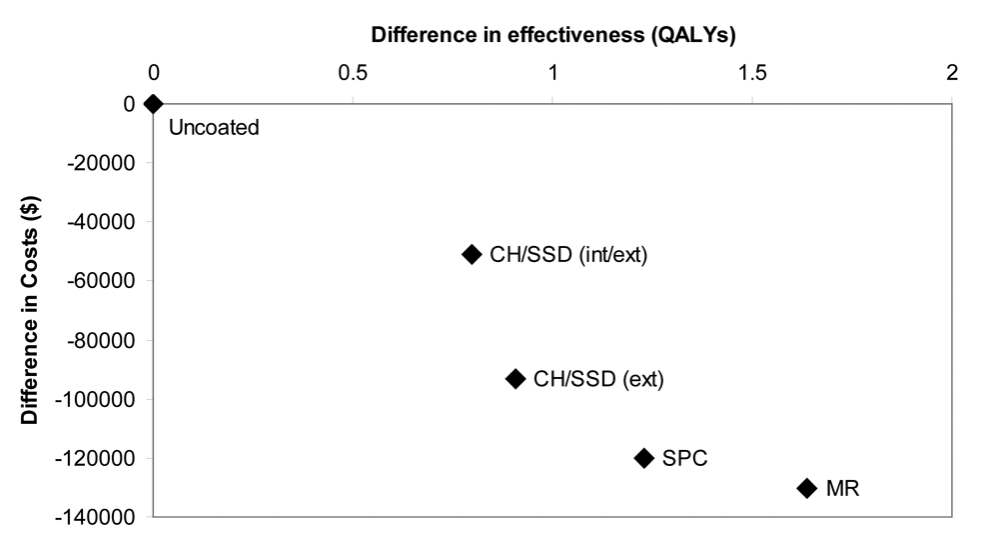
Cost effectiveness of antimicrobial central venous catheters in the baseline analysis (results per 1,000 catheters). CH/SSD (int/ext) = internally and externally coated chlorhexidine and silver sulfadiazine catheters; CH/SSD (ext) = externally coated chlorhexidine and silver sulfadiazine catheters; SPC = silver, platinum and carbon impregnated catheters; MR = minocycline and rifampicin coated catheters; QALY = quality-adjusted life year.

The results of the first analysis, without uncertainty, showed all four types of A-CVC were cost-saving relative to uncoated catheters (Figure 2). The antibiotic-coated catheter (MR) achieved the greatest health benefits and lowest costs and dominated the use of uncoated and the three antiseptic-coated catheter types (SPC, CH/SSD (ext) and CH/SSD (int/ext)). Compared to uncoated catheters, the use of MR catheters avoided 15 infections and generated 1.6 QALYs per 1,000 catheters placed. The MR catheters also released 32 ICU bed days and 95 general ward bed days and achieved cost savings of AUD $130,000 per 1,000 catheters (Table 3).

**Table 3:** Economic evaluation of antimicrobial central venous catheters: incremental costs and health outcomes under baseline analysis

**Catheter type**	**Infections avoided^a^**	**ICU bed days released^a^**	**Costs saved^a ^(2006 AUD)**	**QALYs gained^a^**	**ICER**	**Average monetary net benefits^b ^(95% confidence interval)**
Uncoated						390,664 (371,984 to 408,416)
CH/SSD (ext)	8.4	18.1	$93,281	0.91	Dominated	391,212 (372,736 to 408,687)
CH/SSD (int/ext)	7.4	15.9	$51,126	0.80	Dominated	391,030 (372,467 to 408,574)
SPC	11.4	24.6	$120,062	1.23	Dominated	391,206 (372,687 to 408,772)
MR	15.2	32.8	$130,289	1.64	Cost-saving	391,612 (373,159 to 408,861)

^a^Results presented per 1,000 catheters; ^b^Monetary net benefits reported per catheter assuming a willingness-to-pay for a QALY of AUD $40,000.

CH/SSD, chlorhexidine silver sulfadiazine; ICER, incremental cost effectiveness ratio; ICU, intensive care unit; int/ext, internally and externally coated; QALY, quality-adjusted life year; MR, minocycline and rifampicin; SPC, silver, platinum and carbon.

**Figure 3:**
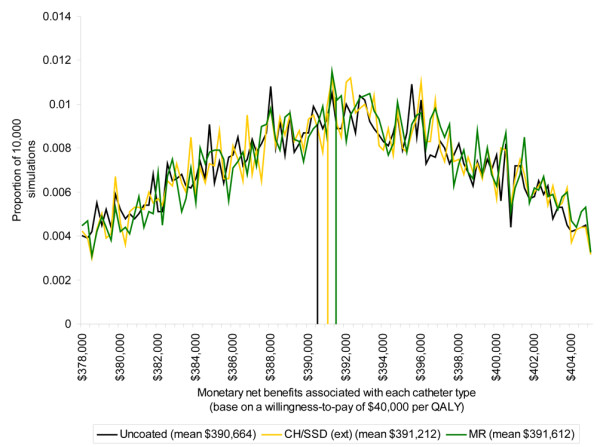
Distribution of monetary net benefits associated with selected catheter types. CH/SSD (ext) = externally coated chlorhexidine and silver sulfadiazine catheters; MR = minocycline and rifampicin coated catheters; QALY = quality-adjusted life year.

The second analysis based on PSA to incorporate uncertainty indicated that at a willingness-to-pay threshold of AUD $40,000 per QALY the average monetary net benefits estimated for each catheter type were very similar with substantial overlap in the 95% CIs (Table 3). Figure 3 shows the distribution of monetary net benefits for the MR, CH/SSD (ext) and uncoated catheters, for clarity the other catheter types have been omitted as their distributions lie over those presented. The MR catheters returned the highest monetary net benefits and represented the optimal choice given current information. They are associated with expected incremental monetary net benefits of AUD $948 per catheter relative to retaining the uncoated type (that is, the average monetary net benefits for a MR catheter, AUD $391,612, minus those for an uncoated catheter, AUD $390,664); however the probability of error in this decision is 62% (Table 4).

**Table 4:** Optimal choice of catheter under uncertainty, given different data scenarios

**Scenario**	**Optimal catheter choice**		**Incremental outcomes with no uncertainty**		**Average incremental monetary net benefits given uncertainty^b^**	
	
	**Catheter type**	**Probability of error**	**Costs**^a^	**QALYs**^a^	**Mean**	**95% Confidence interval**
Baseline	MR	0.62	- $130,289	1.64	948	-106 to 3,792
Low bed day ($335 ICU/$101 ward)	MR	0.76	+ $28,257	1.64	191	-348 to 1,317
High mortality (15%)	MR	0.46	- $106,223	26.65	2,441	116 to 8,516
No utility weights used	MR	0.62	- $130,289	2.33	1,042	-166 to 3,961
Increased length of stay (6.5 days ICU/6 days ward)	MR	0.56	- $282,038	1.64	1,239	-57 to 4,795
Low infection rate (0.8%)	MR	0.75	- $1,972	0.53	325	-71 to 1,283
High infection rate (5.0%)	MR	0.56	- $314,400	3.23	1,725	-139 to 6,455

^a^Relative to uncoated catheters and per 1,000 catheters; ^b^Monetary net benefits reported per catheter relative to uncoated catheters assuming a willingness-to-pay for a QALY of AUD $40,000.

ICU, intensive care unit; QALY, quality-adjusted life year.

For the third analysis, using alternate scenarios for key parameters, the MR catheters maximized monetary net benefits in all cases. However, the probability of error in this decision was consistently high and the 95% CIs for the estimated monetary net benefits associated with these catheters were large (Table 4). The lowest estimate of monetary net benefits arose when bed days were valued at variable cost savings only (that is, the narrow hospital perspective). In this scenario, monetary net benefits associated with MR catheters would be just under AUD $200 per catheter relative to uncoated catheters because the catheters were no longer cost saving instead requiring an increase in expenditure to produce health benefits (Table 4). The highest estimate of monetary net benefits was obtained when an attributable mortality of 15% was assumed. In this scenario, MR catheters were cost-saving and generated high monetary net benefits of nearly AUD $2,441 per catheter relative to retaining uncoated catheters. Scenarios that returned higher estimates of expected monetary net benefits were associated with less uncertainty. Under the high mortality scenario the probability that choosing MR catheters as optimal was incorrect fell to 46%, whilst in the low bed days scenario, where expected monetary net benefits were low, the error probability in this decision was 76%.

## Discussion

Our evaluation suggests any decision regarding the use of A-CVCs in ICU patients is uncertain. The findings from the first analysis, which do not consider uncertainty, concur with existing economic evidence [6,7]. This shows that, for all four types of antimicrobial catheter, health gains will be accompanied by cost savings. Given the assumption of a low attributable mortality and a low rate of infection, expected health gains are minimal and the decision is driven by the change in costs. Most of these costs represent the value of obtaining increased capacity within the ICU, rather than cash savings. Nevertheless, the results of the first analysis imply a decision not to adopt these catheters will harm patients by reducing their health status and increasing their risk of mortality and, simultaneously, waste resources within the healthcare system.

Our second analysis, using PSA, introduces the uncertainty associated with the decision. Based on current information, the MR catheters are the optimal decision because they return the highest net monetary benefits relative to all other catheter types. However, the probability of error in this conclusion is high, at 62%. Our third analysis shows that MR catheters remain the optimal decision across a range of scenarios and quantifies how uncertainty in this decision varies. Uncertainty is lower for scenarios where decision makers believe that attributable mortality is high, where they value bed days highly, or where the starting infection rate is high. This finding fits with conclusions from a recent meta-analysis that suggests that antimicrobial catheters will return a higher treatment benefit when infection rates are high [40], and provides support for current guidelines which recommend reserving their use for settings with high infection rates [9]. However, even in these scenarios the probability that this conclusion is wrong, and the MR catheters are not optimal, does not reduce below 46%.

Interpreting the results of cost effectiveness analyses under uncertainty requires decision makers to think beyond conventional error rates as used in statistical analysis. Decision makers looking to maximize health returns from their budget should choose between these catheters by selecting the option with the highest monetary net benefits. Given the current evidence, MR catheters should be chosen even if the probability of error in this conclusion exceeds the standard level of 5% used to define statistical significance. The justification is that a decision not to use them in favor of uncoated catheters would impose economic costs, arising from average monetary net benefits foregone, of AUD $948 per catheter [41] (AUD $391,612 minus AUD $390,664). This conclusion should lead to rapid and sustained uptake of the technology [5], yet their use appears to be limited despite earlier estimates of these catheters being cost effective [6,7]. We suggest that uncertainty over this cost effectiveness evidence may be partly responsible.

Studies have shown that decision makers are heavily influenced by uncertainty [35,42]. Presenting decision makers with an estimate of uncertainty in the results of an economic evaluation is important for the following reasons: it makes the current state of knowledge about the decision explicit and quantifies confidence (or lack of) in conclusions; it allows them to weigh the cost effectiveness results against other relevant considerations in the adoption decision including their own attitude to risk; and it provides an indication of the value of conducting further research to reduce uncertainty.

Two aspects to uncertainty are important: the probability of making the wrong choice and the potential consequences of getting it wrong. Both elements are required; a decision with a 5% probability of being wrong may still be perceived as uncertain if the consequences are very large. Decision makers tend to be risk averse. Rather than being focused solely on maximizing health returns, they are also concerned with interventions that have the potential to result in harmful outcomes. If there is no potential for harm then decision makers may be happy to accept a new intervention with a high but uncertain benefit. But where the potential for harm is perceived to be high, an existing intervention with a lower benefit may be preferred. Antimicrobial catheters are perceived to carry a risk of a number of negative outcomes that are likely to deter from their introduction, including the potential for a loss of focus on hygiene procedures. There has also been discussion [43] that MR catheters may select for resistant organisms, with higher morbidity and costs [44]. This negative could outweigh potential short-term benefits from these catheters [45]. An absence of clear evidence [46] makes it difficult to quantitatively incorporate this risk into an economic evaluation [47] but it is an important consideration in the adoption decision.

Decision makers deciding whether to use antimicrobial catheters also have a second choice: whether to collect more information to reduce uncertainty in their choice [48]. Value of information analysis [41] can be used to estimate the expected monetary net benefits arising from collecting new information and compare this to the anticipated research costs to indicate whether the research is justifiable. It has been suggested further trials of antimicrobial catheters should be undertaken [43]. Due to the relative rarity of infection these will require a large sample size and the involvement of multiple institutions [43], making them an expensive proposition. Estimating the expected monetary net benefits from a trial would indicate if this is the best way to spend research dollars.

Some important sources of uncertainty have been explored in these analyses, but there are other uncertain elements in this decision that have not been explicitly examined. There is evidence the relative effectiveness of A-CVCs, as compared to uncoated catheters, varies according to duration of catheterization [49] and causative organism [50], and there have been reports of toxicity associated with use of particular types of catheter [13]. However, a lack of data about these concerns both generally, and in relation to each specific coating, meant we were unable to model their impact. If these aspects reduce the effectiveness of any of the catheter types then its cost effectiveness would also be reduced. Alternatively there may be specific subgroups of patients for whom the cost effectiveness of these catheters can be determined with greater certainty. We did not test assumptions about life expectancy and quality of life in ICU survivors, although these will not alter conclusions about which catheter is optimal as all types will be affected equally.

This evaluation, like those reported in earlier studies, is based on a simplified version of a complex decision. It did not include intangible benefits to reduced infection rates, including the increases to clinical morale and public confidence in the healthcare system demonstrated by the national campaigns to reduce rates of CR-BSI [2] and forming part of the rationale for the introduction of the Deficit Reduction Act [4]. Decision makers often consider a wider range of outcomes when deciding on the adoption of a new technology [35,42] and clearly the economic value in reducing infection rates goes beyond the capacity released within hospitals. Valuing these intangible outcomes may improve the representation of the economics of preventing infection, but it would be difficult to achieve. It has been suggested that MR-coated catheters are difficult to insert [6], making them unpopular amongst clinicians, but data comparing failure rates for insertion are not available in order to incorporate this cost. Finally, recent research has shown that improving catheter care by intervention 'bundles' is a highly effective way to reduce rates of CR-BSI [51]. In an evaluation comparing 'bundles' with antimicrobial catheters, it may be that the former would dominate. This is not evaluated here and deserves rigorous exploration rather than hypothesizing.

## Conclusions

Antimicrobial catheters have been available as a means of preventing CR-BSI in the ICU for two decades. Although earlier studies have indicated these devices are cost saving, the findings of this evaluation represent a deeper analysis of the decision than previously available that will help decision makers in any setting considering adopting A-CVCs judge the cost effectiveness of these devices. We have shown that the cost effectiveness of these catheters is uncertain, and are not surprised that infection control decision makers are reticent about using antimicrobial catheters despite the economic evidence. Failure to consider uncertainty generates overly simplistic results and creates skepticism amongst decision makers using them to guide infection control policy. Value of information analyses may suggest where research to reduce this uncertainty should focus, but in the meantime, legislation based on the economics of infection control would do well to consider the complexity of producing good quality evidence in this area.

## Key messages

• The cost effectiveness of antimicrobial catheters to prevent catheter-related bloodstream infection in the intensive care unit is highly uncertain.

• Estimates of cost effectiveness which do not consider uncertainty indicate that minocycline and rifampicin catheters generate the greatest health benefits (1.6 quality-adjusted life years) and opportunity cost savings (AUD $130,289 per 1,000 catheters placed) relative to uncoated catheters.

• When uncertainty in information currently available to inform this decision is considered, MR-coated catheters return the highest monetary net benefits relative to all other catheter types but there is a 62% probability that this is incorrect.

• Value of information analyses may suggest where research to reduce uncertainty in this decision should focus to achieve maximum benefit, but in the meantime, decision makers would do well to consider the complexity of producing good quality evidence in this area.

## Abbreviations

A-CVC: antimicrobial central venous catheter; CR-BSI: catheter-related bloodstream infection; CH/SSD (ext): chlorhexidine/silver sulfadiazine (external coating); CH/SSD (int/ext): chlorhexidine/silver sulfadiazine (internal/external coating); MR: minocycline and rifampicin; QALY: quality-adjusted life year; PSA: probabilistic sensitivity analysis; SPC: silver, platinum and carbon.

## Competing interests

DP has received grant support from AstraZeneca and is a consultant to Three Rivers Pharmaceuticals, Merck, Pfizer, AstraZeneca, Johnson and Johnson, and Sanofi Aventis.

## Authors' contributions

KH coordinated the overall design of the study, collected and analyzed the data and drafted the manuscript. DC aided in defining the clinical context, design of the decision model and collection of data. MW helped conceive of the study and obtain funding. DP aided in defining the clinical context and helped to draft the manuscript. NG conceived of the study and obtained funding, participated in its design and helped to draft the manuscript. All authors read and approved the final manuscript.

## Supplementary Material

Additional file 1 A Word file that provides bibliographic details for all relevant studies retrieved in literature searches for: estimates of the clinical and economic outcomes of CR-BSI, systematic reviews of the effectiveness of antimicrobial catheters, and QALY weights for ICU patients. Click here for file

## References

[B1] Blot SI, Depuydt P, Annemans L, Benoit D, Hoste E, De Waele JJ, Decruyenaere J, Vogelaers D, Colardyn F, Vandewoude KH (2005). Clinical and economic outcomes in critically ill patients with nosocomial catheter-related bloodstream infections. Clin Infect Dis.

[B2] Berwick DM, Calkins DR, McCannon CJ, Hackbarth AD (2006). The 100,000 Lives Campaign: setting a goal and a deadline for improving health care quality. JAMA.

[B3] Safer systems – Saving lives. http://www.health.vic.gov.au/sssl/.

[B4] Centers for Medicare & Medicaid Services (2008). Medicare Program: Changes to the hospital inpatient prospective payment systems and fiscal year 2009 rates; payments for graduate medical education in certain emergency situations; changes to disclosure of physician ownership in hospitals and physician self-referral rules; updates to the long-term care prospective payment system; updates to certain IPPS-excluded hospitals; and collection of information regarding financial relationships between hospitals; final rule. Fed Regist.

[B5] Graves N, Halton KA, Lairson D (2007). Economics and preventing hospital-acquired infection – broadening the perspective. Infect Control Hosp Epidemiol.

[B6] Marciante KD, Veenstra DL, Lipsky BA, Saint S (2003). Which antimicrobial impregnated central venous catheter should we use? Modeling the costs and outcomes of antimicrobial catheter use. Am J Infect Control.

[B7] Shorr AF, Humphreys CW, Helman DL (2003). New choices for central venous catheters. Chest.

[B8] Halton KA, Graves N (2007). Economic evaluation and catheter-related bloodstream infections. Emerg Infect Dis.

[B9] Centers for Disease Control and Prevention (2002). Guidelines for the prevention of intravascular catheter-related infections. Morb Mortal Wkly Rep.

[B10] Bolz K, Ramritu P, Halton K, Cook D, Graves N (2008). Management of central venous catheters in adult intensive care units in Australia: policies and practices. Healthcare Infect.

[B11] Krein SL, Hofer TP, Kowalski CP, Olmsted RN, Kauffman CA, Forman JH, Banaszak-Holl J, Saint S (2007). Use of central venous catheter-related bloodstream infection prevention practices by US hospitals. Mayo Clin Proc.

[B12] Aurich E, Borgert J, Butler M, Cadwallader H, Collignon PJ, Eades M, Fergurson J, Kampen R, Looke D, MacBeth D, McLaws ML, Olesen D, Pawsey M, Richards M, Riley T, Sykes P, Whitby M, West R, Zerner L (2000). Introduction to Australian surveillance definitions: surgical site infections and bloodstream infections. Aust Infect Control.

[B13] Oda T, Hamasaki J, Kanda N, Mikami K (1997). Anaphylactic shock induced by an antiseptic-coated central venous [correction of nervous] catheter. Anesthesiology.

[B14] Brouwer W, Rutten FFH, Koopmanschap M, Drummond M, McGuire A (2001). Costing in economic evaluations. Economic evaluation in health care: merging theory with practice.

[B15] Briggs AH, O'Brien BJ (2001). The death of cost-minimization analysis?. Health Econ.

[B16] Faculty of Intensive Care Australian and New Zealand College of Anaesthetists (1997). Minimum Standards for Intensive Care Units. Policy Document IC-1.

[B17] Mullaney D (2008). Use of variables immediately prior to ICU admission to determine short-term outcomes and assess ICU performance after cardiac surgery. Presented at the Second International Conference on Quality Audit and Outcomes Research in Intensive Care; Christchurch, New Zealand.

[B18] Martin J, Anderson T, Turton C, Hart GK, Hicks P (2006). Intensive Care Resources & Activity: Australia & New Zealand 2003–2005.

[B19] McLaws ML, Berry G (2005). Nonuniform risk of bloodstream infection with increasing central venous catheter-days. Infect Control Hosp Epidemiol.

[B20] Morton AP, Clements AC, Doidge S, Stackelroth J, Curtis M, Whitby M (2008). Surveillance of healthcare-acquired infections in Queensland, Australia: data and lessons from the first 5 years. Infect Control Hosp Epidemiol.

[B21] Ramritu P, Halton KA, Collignon PJ, Cook D, Fraenkel D, Battistutta D, Whitby M, Graves N (2008). A systematic review comparing the relative effectiveness of antimicrobial-coated catheters in intensive care units. Am J Infect Control.

[B22] Warren DK, Quadir WW, Hollenbeak CS, Elward AM, Cox MJ, Fraser VJ (2006). Attributable cost of catheter-associated bloodstream infections among intensive care patients in a nonteaching hospital. Crit Care Med.

[B23] Samore M, Harbarth S, Mayhall CG (2004). A methodologically focused review of the literature in hospital epidemiology and infection control. Hospital Epidemiology and Infection Control.

[B24] Williams TA, Dobb GJ, Finn JC, Knuiman M, Lee KY, Geelhoed E, Webb SA (2006). Data linkage enables evaluation of long-term survival after intensive care. Anaesth Intensive Care.

[B25] Australian Institute of Health and Welfare (2006). Australia's Health 2006. AIHW cat. no. AUS 73.

[B26] Dowdy DW, Eid MP, Sedrakyan A, Mendez-Tellez PA, Pronovost PJ, Herridge MS, Needham DM (2005). Quality of life in adult survivors of critical illness: a systematic review of the literature. Intensive Care Med.

[B27] Hawthorne G, Osborne R (2005). Population norms and meaningful differences for the Assessment of Quality of Life (AQoL) measure. Aust N Z J Public Health.

[B28] Cuthbertson BH, Scott J, Strachan M, Kilonzo M, Vale L (2005). Quality of life before and after intensive care. Anaesthesia.

[B29] Hawthorne G, Richardson J, Day NA (2001). A comparison of the Assessment of Quality of Life (AQoL) with four other generic utility instruments. Ann Med.

[B30] Bureau of Labor Statistics Consumer Price Index. http://www.bls.gov/cpi.

[B31] Rechner IJ, Lipman J (2005). The costs of caring for patients in a tertiary referral Australian intensive care unit. Anaesth Intensive Care.

[B32] Graves N, Birrell FA, Whitby M (2005). Modeling the economic losses from pressure ulcers among hospitalised patients in Australia. Wound Repair Regen.

[B33] Chen Y, Wang F, Liu C, Chou P (2009). Incidence rate and variable cost of nosocomial infections in different types of intensive care units. Infect Control Hosp Epidemiol.

[B34] Kahn JM, Rubenfeld GD, Rohrbach J, Fuchs BD (2008). Cost savings attributable to reductions in intensive care unit length of stay for mechanically ventilated patients. Med Care.

[B35] George B, Harris A, Mitchell A (2001). Cost-effectiveness analysis and the consistency of decision making: evidence from pharmaceutical reimbursement in Australia (1991 to 1996). Pharmacoeconomics.

[B36] Briggs AH (2000). Handling uncertainty in cost-effectiveness models. Pharmacoeconomics.

[B37] Briggs AH, O'Brien BJ, Blackhouse G (2002). Thinking outside the box: recent advances in the analysis and presentation of uncertainty in cost-effectiveness studies. Annu Rev Public Health.

[B38] Cooper NJ, Coyle D, Abrams KR, Mugford M, Sutton AJ (2005). Use of evidence in decision models: an appraisal of health technology assessments in the UK since 1997. J Health Serv Res Policy.

[B39] Coyle D, Lee KM, Donaldson C, Mugford M, Vale L (2002). Evidence-based economic evaluation: how the use of different data sources can impact results. Evidence-based health economics: from effectiveness to efficiency in systematic review.

[B40] Niel-Weise BS, Stijnen T, Broek PJ van der (2007). Anti-infective-treated central venous catheters: a systematic review of randomized controlled trials. Intensive Care Med.

[B41] Claxton K (1999). The irrelevance of inference: a decision making approach to the stochastic evaluation of health care technologies. J Health Econ.

[B42] Devlin N, Parkin D (2004). Does NICE have a cost-effectiveness threshold and what other factors influence its decisions? A binary choice analysis. Health Econ.

[B43] McConnell SA, Gubbins PO, Anaissie EJ (2003). Do antimicrobial-impregnated central venous catheters prevent catheter-related bloodstream infection?. Clin Infect Dis.

[B44] Cosgrove SE, Sakoulas G, Perencevich EN, Schwaber MJ, Karchmer AW, Carmeli Y (2003). Comparison of mortality associated with methicillin-resistant and methicillin-susceptible *Staphylococcus aureus *bacteremia: a meta-analysis. Clin Infect Dis.

[B45] Coast J, Smith RD, Karcher A, Wilton P, Millar MR (2002). Superbugs II: how should economic evaluation be conducted for interventions which aim to contain antimicrobial resistance?. Health Econ.

[B46] Falagas ME, Fragoulis K, Bliziotis IA, Chatzinikolaou I (2007). Rifampicin-impregnated central venous catheters: a meta-analysis of randomized controlled trials. J Antimicrob Chemother.

[B47] Coast J, Smith RD, Millar MR (1996). Superbugs: should antimicrobial resistance be included as a cost in economic evaluation?. Health Econ.

[B48] Claxton K, Sculpher M, Drummond M (2002). A rational framework for decision making by the National Institute for Clinical Excellence. Lancet.

[B49] Geffers C, Zuschneid I, Eckmanns T, Rüden H, Gastmeier P (2003). The relationship between methodological trial quality and the effects of impregnated central venous catheters. Intensive Care Med.

[B50] Leon C, Ruiz-Santana S, Rello J, de la Torre MV, Valles J, Alvarez-Lerma F, Sierra R, Saavedra P, Alvarez-Salgado F (2004). Benefits of minocycline and rifampin-impregnated central venous catheters. Intensive Care Med.

[B51] Pronovost PJ, Needham DM, Berenholtz S, Sinopoli D, Chu H, Cosgrove SE, Sexton B, Hyzy R, Welsh R, Roth G, Bander J, Kepros J, Goeschel C (2006). An intervention to decrease catheter-related bloodstream infections in the ICU. N Engl J Med.

