# Supplementary material for: Cost effectiveness of antimicrobial catheters in the intensive care unit: addressing uncertainty in the decision
Source: Crit Care. 2009 Mar 11;13(2):R35. doi: 10.1186/cc7744 (PMC2689469; doi:10.1186/cc7744)
Supplement: Additional file 1 — A Word file that provides bibliographic details for all relevant studies retrieved in literature searches for: estimates of the clinical and economic outcomes of CR-BSI, systematic reviews of the effectiveness of antimicrobial catheters, and QALY weights for ICU patients. [file cc7744-S1.doc]

ADDITIONAL DATA FILE 1

Cost-effectiveness of antimicrobial catheters in the intensive care unit: addressing uncertainty in the decision

Kate A Halton^1,2^; David A Cook^3^; Michael Whitby^4^; David L Paterson^1,5^; and Nicholas Graves^1,2^

^1^ The Centre for Healthcare Related Infection Surveillance & Prevention, GPO Box 48, Brisbane, Queensland, 4001 Australia

^2^ Institute of Health & Biomedical Innovation, Queensland University of Technology, 60 Musk Avenue, Kelvin Grove, Queensland, 4059 Australia

^3^ Intensive Care Unit, Princess Alexandra Hospital, 199 Ipswich Road, Woolloongabba, Queensland, 4102 Australia

^4^ Infection Management Services, Princess Alexandra Hospital, 199 Ipswich Road, Woolloongabba, Queensland, 4102 Australia

^5^ University of Queensland, Royal Brisbane & Women’s Hospital, Butterfield Street, Herston, Queensland, 4029 Australia

Bibliographic details for relevant articles retrieved in the literature searches

***Exclusion Criteria***

For all searches articles were excluded under the following criteria: no original data; published in a language other than English; undertaken in paediatric, immunosuppressed or oncology patients; inclusion of community-acquired infections; focused on specific microorganisms.

***Articles Retrieved on the Outcomes of CR-BSI***

1. Arnow PM, Quimosing EM, Beach M. Consequences of intravascular catheter sepsis. Clin Infect Dis 1993;16(6):778-84.
2. Leibovici L, Shraga I, Drucker M, Konigsberger H, Samra Z, Pitlik SD. The benefit of appropriate empirical antibiotic treatment in patients with bloodstream infection. J Intern Med 1998;244(5):379-86.
3. Byers KE, Adal KA, Anglim AM, Farr BM. Case fatality rate for catheter-related bloodstream infections (CR-BSI): a meta-analysis [abstract 43]. In: 5th Annual Meeting of the Society of Healthcare Epidemiology of America, 1995; San Diego: p. 23.
4. Pittet D, Li N, Woolson RF, Wenzel RP. Microbiological factors influencing the outcome of nosocomial bloodstream infections: a 6-year validated, population-based model. Clin Infect Dis 1997;24(6):1068-78.
5. Charalambous C, Swoboda SM, Dick J, Perl T, Lipsett PA. Risk factors and clinical impact of central line infections in the surgical intensive care unit. Arch Surg 1998;133(11):1241-6.
6. Soufir L, Timsit JF, Mahe C, Carlet J, Regnier B, Chevret S. Attributable morbidity and mortality of catheter-related septicemia in critically ill patients: a matched, risk-adjusted, cohort study. Infect Control Hosp Epidemiol 1999;20(6):396-401.
7. Lark RL, Chenoweth C, Saint S, Zemencuk JK, Lipsky BA, Plorde JJ. Four year prospective evaluation of nosocomial bacteremia: epidemiology, microbiology, and patient outcome. Diagn Microbiol Infect Dis 2000;38(3):131-40.
8. Rello J, Ochagavia A, Sabanes E, Roque M, Mariscal D, Reynaga E, et al. Evaluation of outcome of intravenous catheter-related infections in critically ill patients. Am J Respir Crit Care Med 2000;162(3 Pt 1):1027-30.
9. Garrouste-Orgeas M, Chevret S, Mainardi JL, Timsit JF, Misset B, Carlet J. A one-year prospective study of nosocomial bacteraemia in ICU and non-ICU patients and its impact on patient outcome. J Hosp Infect 2000;44(3):206-13.
10. Siegman-Igra Y, Golan H, Schwartz D, Cahaner Y, De-Mayo G, Orni-Wasserlauf R. Epidemiology of vascular catheter-related bloodstream infections in a large university hospital in Israel. Scand J Infect Dis 2000;32(4):411-5.
11. Pelletier SJ, Crabtree TD, Gleason TG, Pruett TL, Sawyer RG. Bacteremia associated with central venous catheter infection is not an independent predictor of outcomes. J Am Coll Surg 2000;190(6):671-80; discussion 80-1.
12. Dimick JB, Pelz RK, Consunji R, Swoboda SM, Hendrix CW, Lipsett PA. Increased resource use associated with catheter-related bloodstream infection in the surgical intensive care unit. Arch Surg 2001;136(2):229-34.
13. Renaud B, Brun-Buisson C. Outcomes of primary and catheter-related bacteremia. A cohort and case-control study in critically ill patients. Am J Respir Crit Care Med 2001;163(7):1584-90.
14. Vales EC, Abraira V, Sánchez JC, García MP, Feijoo AR, Alvarez MJ, et al. A predictive model for mortality of bloodstream infections. Bedside analysis with the Weibull function. J Clin Epidemiol 2002;55(6):563-72.
15. Rosenthal VD, Guzman S, Orellano PW. Nosocomial infections in medical-surgical intensive care units in Argentina: attributable mortality and length of stay. Am J Infect Control 2003;31(5):291-5.
16. Rosenthal VD, Guzman S, Migone O, Crnich CJ. The attributable cost, length of hospital stay, and mortality of central line-associated bloodstream infection in intensive care departments in Argentina: A prospective, matched analysis. Am J Infect Control 2003;31(8):475-80.
17. Pawar M, Mehta Y, Kapoor P, Sharma J, Gupta A, Trehan N. Central venous catheter-related blood stream infections: incidence, risk factors, outcome, and associated pathogens. J Cardiothoracic Vasc Anesth 2004;18(3):304-8.
18. Blot SI, Depuydt P, Annemans L, Benoit D, Hoste E, De Waele JJ, et al. Clinical and economic outcomes in critically ill patients with nosocomial catheter-related bloodstream infections. Clin Infect Dis 2005;41(11):1591-8.
19. Moreno CA, Rosenthal VD, Olarte N, Gomez WV, Sussmann O, Agudelo JG, et al. Device-associated infection rate and mortality in intensive care units of 9 Colombian hospitals: findings of the International Nosocomial Infection Control Consortium. Infect Control Hosp Epidemiol 2006;27(4):349-56.
20. Shannon RP, Patel B, Cummins D, Shannon AH, Ganguli G, Lu Y. Economics of central line--associated bloodstream infections. Am J Med Qual 2006;21(6 Suppl):7S-16S.
21. Garrouste-Orgeas M, Timsit JF, Tafflet M, Misset B, Zahar JR, Soufir L, et al. Excess risk of death from intensive care unit-acquired nosocomial bloodstream infections: a reappraisal. Clin Infect Dis 2006;42(8):1118-26.
22. Rosenthal VD, Maki DG, Salomao R, Moreno CA, Mehta Y, Higuera F, et al. Device-associated nosocomial infections in 55 intensive care units of 8 developing countries. Ann Intern Med 2006;145(8):582-91.
23. Warren DK, Quadir WW, Hollenbeak CS, Elward AM, Cox MJ, Fraser VJ. Attributable cost of catheter-associated bloodstream infections among intensive care patients in a nonteaching hospital. Crit Care Med 2006;34(8):2084-9.
24. Higuera F, Rangel-Frausto MS, Rosenthal VD, Soto JM, CastaÃ±on J, Franco G, et al. Attributable cost and length of stay for patients with central venous catheter-associated bloodstream infection in Mexico City intensive care units: a prospective, matched analysis. Infect Control Hosp Epidemiol 2007;28(1):31-5.
25. van der Kooi TI, de Boer AS, Manniën J, Wille JC, Beaumont MT, Mooi BW, et al. Incidence and risk factors of device-associated infections and associated mortality at the intensive care in the Dutch surveillance system. Intens Care Med 2007;33(2):271-8.

***Reviews of Antimicrobial-Coated Catheters***

1. Haxhe JJ, D'Hoore W. A meta-analysis dealing with the effectiveness of chlorhexidine and silver-sulfadiazine impregnated central venous catheters. J Hosp Infect 1998;40:166-8.
2. Veenstra DL, Saint S, Saha S, Lumley T, Sullivan SD. Efficacy of antiseptic-impregnated central venous catheters in preventing catheter-related bloodstream infection: a meta-analysis. JAMA 1999;281:261-7.
3. Mermel LA. Prevention of intravascular catheter-related infections. Ann Intern Med 2000;132(5):391-402.
4. Marin MG, Lee JC, Skurnick JH. Prevention of nosocomial bloodstream infections: effectiveness of antimicrobial-impregnated and heparin-bonded central venous catheters. Crit Care Med 2000;28(9):3332-8.
5. Donelli G, Francolini I. Efficacy of antiadhesive, antibiotic and antiseptic coatings in preventing catheter-related infections: review. J Chemother 2001;13(6):595-606.
6. Pai MP, Pendland SL, Danziger LH. Antimicrobial-coated/bonded and -impregnated intravascular catheters. Ann Pharmacother 2001;35:1255-63.
7. Walder B, Pittet D, Tramèr MR. Prevention of bloodstream infections with central venous catheters treated with anti-infective agents depends on catheter type and insertion time: evidence from a meta-analysis. Infect Control Hosp Epidemiol 2002;23(12):748-56.
8. McConnell SA, Gubbins PO, Anaissie EJ. Do antimicrobial-impregnated central venous catheters prevent catheter-related bloodstream infection? Clin Infect Dis 2003;37(1):65-72.
9. Geffers C, Zuschneid I, Eckmanns T, Rüden H, Gastmeier P. The relationship between methodological trial quality and the effects of impregnated central venous catheters. Intensive Care Med 2003;29(3):403-9.
10. Gastmeier P, Zuschneid I, Geffers C. Antimicrobially impregnated catheters: an overview of randomised controlled trials. J Vasc Access 2003;4:102-10.
11. Sherertz RJ. Update on vascular catheter infections. Curr Opin Infect Dis 2004;17:303-7.
12. Falagas ME, Fragoulis K, Bliziotis IA, Chatzinikolaou I: Rifampicin-impregnated central venous catheters: a meta-analysis of randomized controlled trials. J Antimicrob Chemother 2007, 59:359-369.
13. Neil-Weise BS, Stijnen T, van der Broek PJ. Anti-infective-treated central venous catheters: a systematic review of randomized controlled trials. Intensive Care Med 2007;33(12):2058-68.
14. Ramritu P, Halton KA, Collignon PJ, Cook D, Fraenkel D, Battistutta D, et al. A systematic review comparing the relative effectiveness of antimicrobial-coated catheters in intensive care units. Am J Infect Control 2008;36(2):104-17.

***Estimates of QALY weights for intensive care unit patients***

Review Articles

1. Tengs TO, Wallace A. One thousand health-related quality-of-life estimates. Med Care 2000;38:583-637.
2. Chaboyer W, Elliott D. Health-related quality of life of ICU survivors: review of the literature. Intensive Crit Care Nurs 2000;16(2):88-97.
3. American Thoracic Society. Understanding costs and cost-effectiveness in critical care. Am J Respir Crit Care Med 2002;165(4):540-50.
4. Dowdy DW, Eid MP, Sedrakyan A, Mendez-Tellez PA, Pronovost PJ, Herridge MS, et al. Quality of life in adult survivors of critical illness: a systematic review of the literature. Intensive Care Med 2005;31(5):611-20.
5. Talmor D, Shapiro N, Greenberg D, Stone PW, Neumann PJ. When is critical care medicine cost-effective? A systematic review of the cost-effectiveness literature. Crit Care Med 2006 Nov;34(11):2738-47.
6. Ridley S, Morris S. Cost-effectiveness of adult intensive care in the UK. Anaesthesia 2007 Jun;62(6):547-54.

Original Articles

- 1. Danis M, Patrick DL, Southerland LI, Green ML. Patient's and families' preferences for medical intensive care. JAMA 1988;260:797-802.
  2. Patrick DL, Danis M, Southerland LI, Hong G. Quality of life following intensive care. J Gen Intern Med 1988;3:218-223.
  3. Ridley S, Biggam M, Stone P. A cost-utility analysis of intensive therapy. II. Quality of life in survivors. Anaesthesia 1994;49:192–6.
  4. Kerridge RK, Glasziou PP, Hillman KM. The use of “quality-adjusted life years” (QALYs) to evaluate treatment in intensive care. Anaesth Intens Care 1995;23:322-31.
  5. Badia X, Díaz-Prieto A, Rué M, Patrick DL. Measuring health and health state preferences among critically ill patients. Intensive Care Med 1996;22(12):1379-84.
  6. Ridley SA, Chrispin P, Scotton H, Rogers J, Lloyd D. Changes in quality of life after intensive care: Comparison with normal data. Anaesthesia 1997;52:195–202.
  7. Eddleston JM, White P, Guthrie E. Survival, morbidity, and quality of life after discharge from intensive care. Crit Care Med 2000;28:2293–9.
  8. Sznajder M, Aegerter P, Launois R, Merliere Y, Guidet B, CubRea. A cost-effectiveness analysis of stays in intensive care units. Intensive Care Med 2001;27(1):146-53.
  9. Badia X, Diaz-Prieto A, Gorriz MT, Herdman M, Torrado H, Farrero E, Cavanilles JM. Using the EuroQol-5D to measure changes in quality of life 12 months after discharge from an intensive care unit. Intensive Care Med 2001;27(12):1901-7.
  10. Granja C, Teixeira-Pinto A, Costa-Pereira A. Quality of life after intensive care--evaluation with EQ-5D questionnaire. Intensive Care Med 2002;28(7):898-907.
  11. García Lizana F, Peres Bota D, De Cubber M, Vincent JL. Long-term outcome in ICU patients: what about quality of life? Intensive Care Med 2003;29(8):1286-93.
  12. Elliott D, Mudaliar Y, Kim C. Examining discharge outcomes and health status of critically ill patients: some practical considerations. Intensive Crit Care Nurs 2004;20(6):366-77.
  13. Cuthbertson BH, Scott J, Strachan M, Kilonzo M, Vale L. Quality of life before and after intensive care. Anaesthesia 2005;60:332–9.
  14. Graf J, Wagner J, Graf C, Koch KC, Janssens U. Five-year survival, quality of life, and individual costs of 303 consecutive medical intensive care patients--a cost-utility analysis. Crit Care Med 2005;33(3):547-55.
